# The Tol-Pal system of *Acinetobacter baumannii* is important for cell morphology, antibiotic resistance and virulence

**DOI:** 10.1007/s10123-022-00319-9

**Published:** 2023-01-17

**Authors:** Josephine Joy Hubloher, Lisa Van der Sande, Christoph Schaudinn, Volker Müller, Beate Averhoff

**Affiliations:** 1 Department of Molecular Microbiology & Bioenergetics, Institute of Molecular Biosciences, Goethe-University Frankfurt Am Main, Max-Von-Laue-Str. 9, 60438 Frankfurt, Germany; 2 Advanced Light and Electron Microscopy ZBS4, Robert-Koch-Institut, Berlin, Germany

**Keywords:** Virulence, Tol-Pal system, *Acinetobacter baumannii*, Infection

## Abstract

*Acinetobacter baumannii* is an opportunistic human pathogen that has become a global threat to healthcare institutions. This Gram-negative bacterium is one of the most successful human pathogens worldwide and responsible for hospital-acquired infections. This is due to its outstanding potential to adapt to very different environments, to persist in the human host and most important, its ability to develop multidrug resistance. Our combined approach of genomic and phenotypic analyses led to the identification of the envelope spanning Tol-Pal system in *A. baumannii*. We found that the deletion of the *tolQ*, *tolR*, *tolA*, *tolB*, and *pal* genes affects cell morphology and increases antibiotic sensitivity, such as the ∆*tol*-*pal* mutant exhibits a significantly increased gentamicin and bacitracin sensitivity. Furthermore, *Galleria mellonella* caterpillar killing assays revealed that the ∆*tol*-*pal* mutant exhibits a decreased killing phenotype. Taken together, our findings suggest that the Tol-Pal system is important for cell morphology, antibiotic resistance, and virulence of *A. baumannii*.

## Introduction

The Gram-negative opportunistic human pathogen *Acinetobacter baumannii* is one of the six most important multidrug-resistant nosocomial pathogens worldwide (Dijkshoorn et al. 2007; Antunes et al. 2014; World Health Organization 2017). One key to its success in hospital environments is its ability to persist and survive within hospital settings (Dijkshoorn et al. 2007). In particular, resistance against a broad range of antibiotics is responsible for the spread of *A. baumannii* in health care systems around the world (Dijkshoorn et al. 2007; Göttig et al. 2014). The increase in multidrug or even pan drug resistant *A. baumannii* strains is due to the acquisition of resistance mechanisms such as hydrolyzing enzymes (e.g., β-lactamases) and efflux pumps (Dijkshoorn et al. 2007; Kempf and Rolain 2012; Roca et al. 2012). In addition, there are intrinsic properties such as membrane permeability which affect antibiotic resistances of *A. baumannii*. For example, capsular polysaccharides and the OmpA porin decrease the membrane permeability of *A. baumannii* and thereby decrease susceptibility to antimicrobial agents (Roca et al. 2012; Lee et al. 2017). Analyses of colicin resistant *Escherichia coli* mutants led to the identification of Tol-Pal proteins important for outer membrane integrity and permeability (Kowata et al. 2016; Szczepaniak et al. 2020). Hetero-oligomeric Tol-Pal membrane protein complexes have been found in all clades of proteobacteria and play a role in charge transfer from the inner to the outer membrane and in cell division by remodeling septal peptidoglycan at division sites (Léonard et al. 2022; Webby et al. 2022). The Tol-Pal system is also important for pathogenesis and virulence such as it plays a role in type III secretion, bacterial motility, adhesion to epithelial cells, persister cell survival in the presence of antibiotics, and capsule formation of different pathogenic bacteria (Hirakawa et al. 2020). Information with respect to the Tol-Pal system in *A. baumannii* is very scarce. Here we report on the role of the Tol-Pal system in cell morphology, antibiotic resistance, and virulence of *A. baumannii*.

## Materials and methods

### Bacterial strains and culture conditions

*E. coli* DH5α was grown at 37 °C in LB medium (Bertani 1951). The *A. baumannii* wild type strain ATCC 19606T and the ∆*tol*-*pal* mutant were grown at 37 °C either in LB medium (Bertani 1951), in tryptic soy broth (TSB), or mineral medium with 20 mM succinate as sole carbon and energy source (Zeidler et al. 2017). For solid media, 1.8% agar was added. Growth experiments were started by inoculation of fresh medium with an overnight culture to an initial optical density (OD_600nm_) of 0.1. Growth was monitored photometrically (600_ nm_). Kanamycin (20 µg/ml) or tetracycline (30 µg/ml) was added from stock solutions when appropriate.

### Generating the inserts for RecAB-mediated gene editing

Replacement of the *tol*-*pal* gene cluster by a kanamycin resistance cassette was performed by using the RecAB mediated recombineering system for *A. baumannii* (Tucker et al. 2014). Therefore, the recombinant plasmid pBIISK_∆*tol*-*pal*::*kan*^*R*^ was generated. Approximately 300 bp upstream and downstream of the *tol*-*pal* gene cluster were amplified using the primer pairs up*tolQ*_fwd and up*tolQ*_rev or down*pal*_fwd and down*pal*_rev, respectively (primers are listed in Table 1). A kanamycin resistance cassette flanked by flippase recognition sites (FRT-sites) was amplified from the plasmid pKD4 using the primer pair *kan*^*R*^-FRT_fwd and *kan*^*R*^*-*FRT_rev. The plasmid pBIISK was amplified using the primers pBIISK_fw and pBIISK_rev. The resulting PCR products were assembled by Gibson assembly according to the instructions of the manufacturer (Gibson Assembly Master Mix, New England Biolabs, Ipswich, MA, USA). The resulting recombinant plasmid pBIISK_∆*tol*-*pal*::*kan*^*R*^ was amplified using the primers lppΔ*tolQ*-*pal*_fwd and lppΔ*tolQ*-*pal*_rev. The PCR product was used for replacement of the *tol*-*pal* locus using a RecAB dependent recombineering system.

**Table 1:** Primers used in this study

Primer	Sequence 5′ → 3′
pBIISK_fwd	atcgaattcctgcagccc
pBIISK_rev	atcaagcttatcgataccgtc
up*tolQ*_fwd	acggtatcgataagcttgatccattactggcatcaaaaag
up*tolQ*_rev	cacaatcgctcatagttacatatgccgg
*kan*^*R*^*-*FRT_fwd	tgtaactatgagcgattgtgtaggctgg
*kan*^*R−*^FRT_rev	ggatgttttaatatgaatatcctccttagttcctattc
down*pal*_fwd	atattcatattaaaacatcccaaaaaataaacg
down*pal*_rev	ccgggctgcaggaattcgattttacgtggtatgttgtttg
lppΔ*tolQ-pal*_fwd	tgcttctggtgaggttgag
lppΔ*tolQ-pal*_rev	gattatgaggcaaaacctg
Δ*tolQ-pal*_fwd	acagcagtcgcgattgaaag
Δ*tolQ-pal*_rev	caagtcgcagcaattgtgtc

### RecAB mediated gene replacement

The RecAB mediated gene replacement was performed according to Tucker et al. (2014). Therefore, *A. baumannii* ATCC 19606T was transformed with the plasmid pAT04 encoding the RecAB system using electroporation (2.5 kV, 200 Ω, and 25 μF). *A. baumannii* ATCC 19606T + pAT04 was selected on solid LB medium with tetracycline. The overnight culture was used to inoculate fresh LB medium followed by incubation at 37 °C for 1 h and addition of IPTG to a final concentration of 2 mM. After a further incubation at 37 °C for 3 h, cells were harvested and washed three times with ice-cold H_2_O containing 10% glycerol (50 ml) and finally resuspended in 200 µl H_2_O containing 10% glycerol. One hundred microliters of the resulting cell suspension was mixed with 5 µg of the PCR product and electroporated (2.5 kV, 200 Ω, and 25 μF). The electroporated cells were grown in LB medium with 2 mM IPTG for 4 h, followed by centrifugation and plating onto solid LB medium with kanamycin. Single colonies were obtained after overnight incubation at 37 °C. Replacement of the *tol*-*pal* genes by the FRT-flanked kanamycin resistance cassette led to a ∆*tol*-*pal*::*kan*^*R*^ mutant. Replacement of the *tol-pal* genes was verified via PCR. The plasmid was cured by two transfers of the ∆*tol*-*pal*::*kan*^*R*^ mutant in LB medium without tetracycline.

### FLP mediated removal of the kanamycin resistance cassette

Generation of a markerless ∆*tol*-*pal* mutant was performed by flippase (FLP) mediated removal of the kanamycin cassette using the method of Tucker et al. (2014). Therefore, the ∆*tol*-*pal*::*kan*^*R*^mutant was transformed with pAT03_tet (Breisch et al. 2022) using electroporation (2.5 kV, 200 Ω, and 25 μF). Transformants were selected on solid LB medium in the presence of tetracycline. The resulting strain ATCC 19606T ∆*tol*-*pal*::*kan*^*R*^ + pAT03_tet was grown overnight in LB medium with tetracycline and 0.1 mM IPTG. The overnight culture was plated onto solid LB medium with tetracycline and 0.1 mM IPTG. After overnight incubation at 37 °C, single colonies were obtained and analyzed with respect to kanamycin sensitivity. The ∆*tol*-*pal* mutant was verified via PCR.

### Scanning electron microscopy

The *A. baumannii* wild type strain and the ∆*tol*-*pal* mutant were grown on glass coverslips in 12 well plates in TSB medium at 37 °C for 24 h. Samples were fixed (1.0% paraformaldehyde, 2.5% glutaraldehyde in 50 mM HEPES) for 24 h; dehydrated in 30, 50, 70, 90, 95, 100, and 100% ethanol; critical point dried; mounted on aluminum stubs; sputter coated with a 12 nm layer of gold–palladium; and finally examined in the SEM (ZEISS 1530 Gemini, Carl Zeiss Microscopy GmbH, Germany) operating at 3 kV using the in-lens electron detector.

### Antibiotic and detergent resistance analyses

*A. baumannii* strains were grown overnight in LB medium. Cells were harvested and washed with sterile saline and adjusted to an OD_600nm_ of 1. Serial dilutions were prepared using sterile saline and 4 µl of the cell suspensions was dropped onto solid LB medium containing SDS or different antibiotics. Cells were incubated overnight at 37 °C.

### Human serum killing assay

Analysis of the sensitivity of *A. baumannii* and the Δ*tol*-*pal* mutant to the human complement system was performed as described recently (Breisch et al. 2022). Therefore, overnight cultures of the *A. baumannii* wild type and the ∆*tol*-*pal* mutant were inoculated into fresh LB medium to an initial OD_600nm_ of 0.1. Strains were grown at 37 °C to an OD_600nm_ of 0.5–0.6, harvested, washed twice with sodium phosphate buffer (50 mM; pH 7) containing 0.9 mM CaCl_2_ and 0.5 mM MgCl_2_ (SPB^+^/^+^), and adjusted to an optical density of 0.4. Ten microliters of the cell suspension was added to 190 μl SPB^+^/^+^ with normal human serum concentrations in the range of 0–15%. The suspensions were incubated for 2 h at 37 °C. After incubation, 800 µl SPB^+^/^+^ was added to the samples, serial dilutions were prepared, and colony forming units were determined after growth on solid LB medium.

### LL-37 killing assay

Resistance of *A. baumannii* and the Δ*tol*-*pal* mutant to the human peptide LL-37 was analyzed as described by Lin et al. (2015). Overnight cultures of the *A. baumannii* wild type strain and the ∆*tol*-*pal* mutant were used to inoculate LB medium to an initial OD_600nm_ of 0.1. Strains were grown at 37 °C to an OD_600nm_ of 0.5–0.6, harvested, washed twice with sodium phosphate buffer (50 mM; pH 7), and adjusted to an optical density of 0.2. Ten microliters of the cell suspensions was added to sodium phosphate buffer containing different LL-37 concentrations. The cell suspensions were incubated at 37 °C for 3 h. After incubation, serial dilutions were prepared and the colony forming units were determined after growth on solid LB medium.

### *Galleria mellonella* infection assay

*G. mellonella* infection assays were performed as described recently (Hubloher et al. 2021; König et al. 2021). The *A. baumannii* wild type strain and the ∆*tol*-*pal* mutant were grown in LB medium and harvested in the exponential growth phase (OD_600nm_ = 0.5). Cells were washed and resuspended in sterile saline. For each infection assay, 16 caterpillars were injected with 10 µl cell suspensions (approximately 1*10^6^ CFU) into the last proleg of preselected *G. mellonella* caterpillars (weight range between 0.35 and 0.45 g/larvae). A caterpillar control group was injected with 10 µl sterile saline and another control group was untreated. Caterpillars were incubated for 7 days in the dark at 37 °C. *p* values were determined by using an unpaired *t*-test (GraphPad Prism 6 Software) and *p* values of ≤ 0.05 were considered statistically significant.

## Results

### Tol-Pal homologs in *A. baumannii* ATCC 19606T

**Fig. 1:**
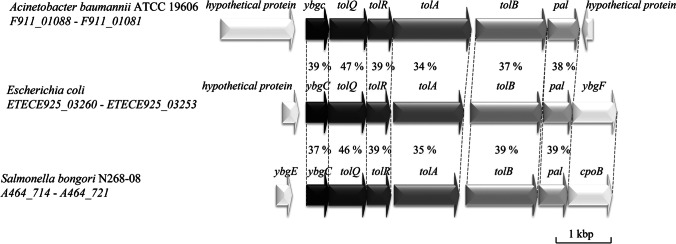
Genetic organization of *tol*-*pal* gene clusters. The amino acid identities between the Tol-Pal (in percent) of *A. baumannii* ATCC 19606T and other Tol-Pal proteins are stated between the distinct gene clusters

We searched the *A. baumannii* ATCC 19606T genome for potential genes of the Tol-Pal system and found genes of the Tol-Pal core system, annotated as *tolQ*, *tolR*, *tolA*, *tolB*, and *pal*. The presence of these core system subunits of the Tol-Pal system is conserved among proteobacteria (Szczepaniak et al. 2020). However, in addition to the conserved core genes some bacteria contain a *ybgC* and/ or a *cpoB* gene up- or downstream of the core gene cluster (Fig. 1). In *A. baumannii*, the *tol*-*pal* core genes are preceded a *ybgC* gene. A *cpoB* gene is not present in this gene cluster and was also not detected in any distant locus in the genome. Downstream of the *tol*-*pal* genes in opposite orientation a gene encoding a hypothetical protein which is not in any functional context with the Tol-Pal system was detected (Fig. 1). The *tol*-*pal* genes and the genetic organization of the *tol*-*pal* gene cluster is conserved in the *Acinetobacter* genus, e.g., in *Acinetobacter baylyi* and species of the *A. baumannii*-*calcoaceticus* complex (*Acinetobacter calcoaceticus*, *A. baumannii*, *Acinetobacter nosocomialis*, *Acinetobacter pittii*, and *Acinetobacter seifertii*). Sequence alignments of the deduced Tol-Pal proteins of *A. baumannii* revealed significant similarities to the Tol-Pal proteins of *E. coli* and *Salmonella bongori*, such as identities in the range of 47–34% were found (Fig. 1). These amino identities together with the conserved organization of the genes of the potential *tol*-*pal* gene cluster suggest that *tolQ*, *tolR*, *tolA*, *tolB*, and *pal* encode the core proteins of the Tol-Pal system in *A. baumannii* ATCC 19606T.

### The Tol-Pal system is important for cell morphology

**Fig. 2:**
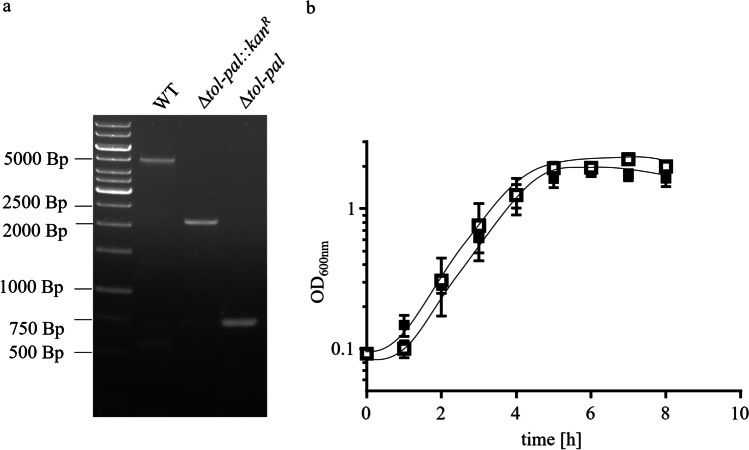
PCR verification of the *tol*-*pal* mutants (**a**) and growth of the wild type and the ∆*tol*-*pal* mutant in mineral medium with succinate (**b**). Precultures of the *A. baumannii* wild type strain (□) and the ∆*tol*-*pal* mutant (■) were grown overnight in mineral medium with succinate and were used to inoculate prewarmed fresh mineral medium to an initial OD_600nm_ of 0.1. Each value is the mean ± SEM of at least three independent experiments

The functionality of the Tol-Pal system of *A. baumannii* in cell morphology was addressed by deleting the genes of the Tol-Pal core system (*tolQ*, *tolR*, *tolA*, *tolB*, and *pal*). RecAB-mediated replacement of *tol*-*pal* locus by a kanamycin resistance cassette led to a ∆*tol*-*pal::kan*^*R*^ mutant (Tucker et al. 2014). Excision of the kanamycin resistant cassette was mediated by the FLP and resulted in a markerless *A. baumannii* ATCC 19606T ∆*tol*-*pal* mutant. The generated mutants ∆*tol*-*pal::kan*^*R*^and ∆*tol*-*pal* were verified by PCR using the primers Δ*tolQ-pal*_fwd and Δ*tolQ-pal*_rev. Amplification of the *tol*-*pal* locus in the wild type resulted in a PCR product with the size of 5172 bp, whereas amplification of the *tol*-*pal* locus from the genome of the ∆*tol*-*pal*::*kan*^*R*^ mutant led to a PCR product of 2123 bp and the PCR product generated with genomic DNA of the ∆*tol*-*pal* mutant comprises of 619 bp (Fig. 2a). These PCR fragments correspond to the expected DNA fragments and confirm that the mutants are correct. A polar effect of the markerless deletion of the *tol*-*pal* genes can be excluded since the gene downstream of the *tol*-*pal* genes is divergently transcribed and the hypothetical protein product is not in any functional context with the Tol-Pal system.

Analysis of the growth phenotype of the ∆*tol*-*pal* mutant revealed that growth in mineral medium was unaffected (Fig. 2b). A comparable growth was also found in LB medium (data not shown).

**Fig. 3:**
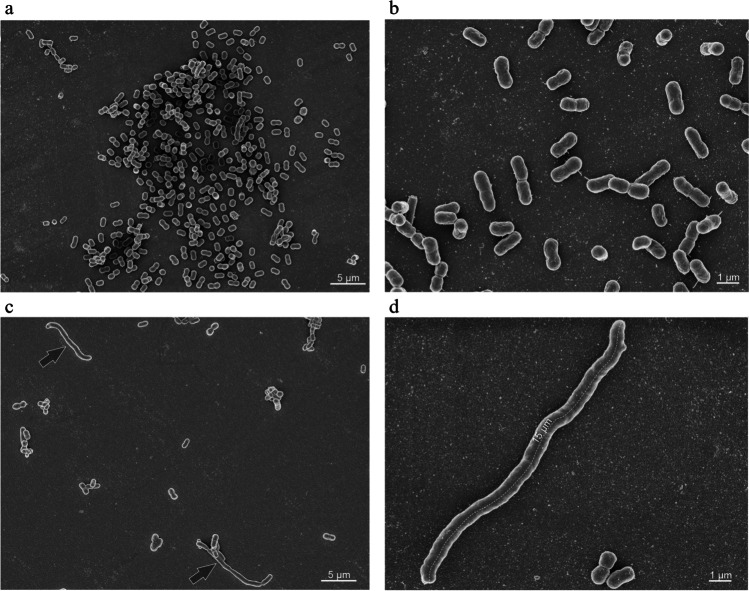
Scanning electron micrographs of the *A. baumannii* wild type strain and the ∆*tol*-*pal* mutant. *A. baumannii* wild type cells and the ∆*tol*-*pal* mutant were grown on glass coverslips in 12 well plates in TSB medium at 37 °C for 24 h. The wild type cells formed typical short rods (**a**, **b**), whereas many mutant cells were found to form cells with extraordinary length (arrows) (**c**, **d**). One out of 50 ∆*tol*-*pal* cells showed an dramatic increase in cell length. The analysis has been done by quantifying 7 randomly picked images containing 50–100 cells. Images **a** and **c** were taken at a magnification of 5,500 fold, and **b** and **d** at 16,000 fold

To analyze the role of the Tol-Pal system in cell morphology of the *A. baumannii* wild type strain and ∆*tol*-*pal* mutant was analyzed by scanning electron microscopy. These studies revealed that many cells of the ∆*tol*-*pal* mutant exhibited an extraordinary length up to twenty-five times of their normal length (Fig. 3c and d). This elongated phenotype was never observed with the wild type strain (Fig. 3a and b). These findings suggest that the Tol-Pal system of *A. baumannii* plays a role in cell morphology.

### The ∆*tol-pal* mutant is impaired in antibiotic and SDS resistance

**Fig. 4:**
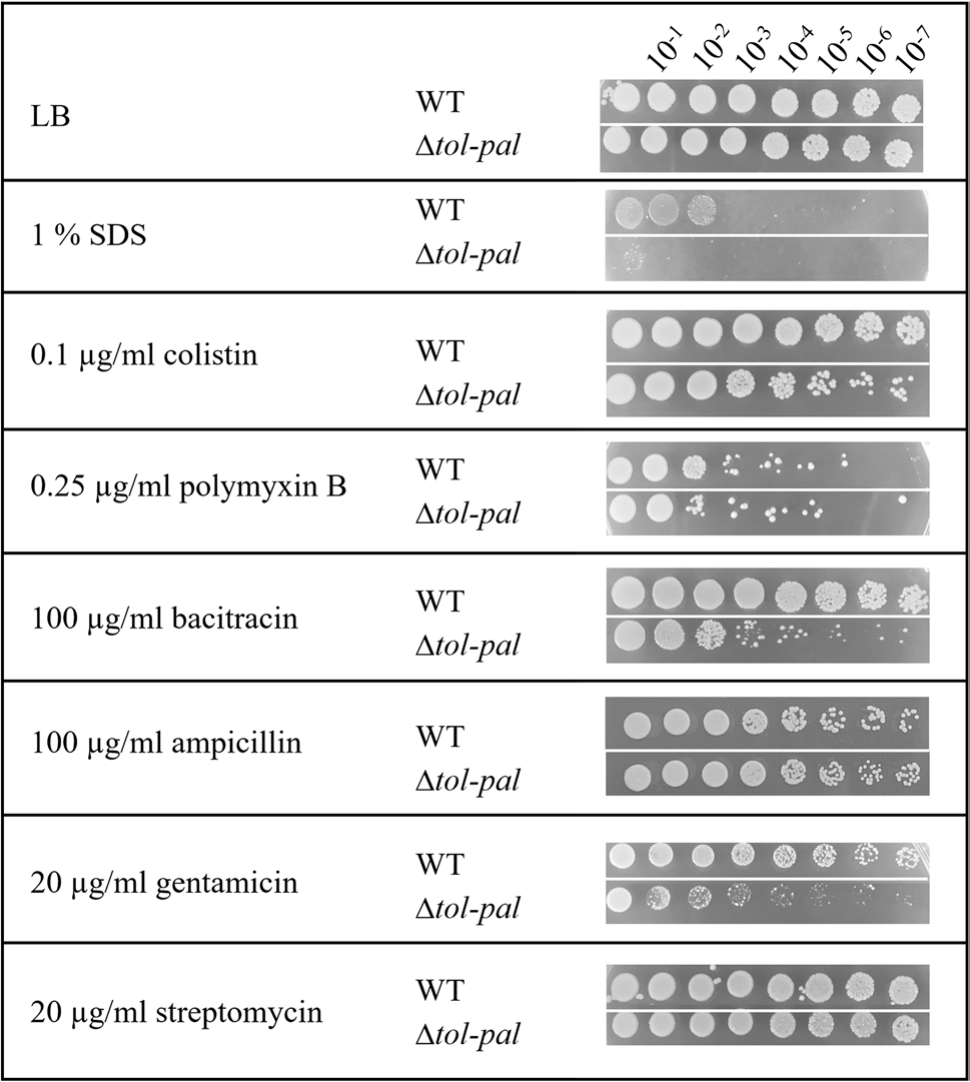
Effect of the *tol*-*pal* deletion on the viability of *A. baumannii* in presence of SDS or antibiotics. Serial dilutions of *A. baumannii* and the ∆*tol*-*pal* mutant were prepared and 4 µl of the cell suspensions was dropped onto solid LB medium containing either SDS or different antibiotics followed by overnight incubation at 37 °C. One representative experiment of at least three independent replicates is shown

The role of the Tol-Pal system in membrane integrity prompted us to analyze the resistance of the ∆*tol-pal* mutant to antibiotics and detergents via drop dilution assays (Fig. 4). Both strains grew comparably up to the highest dilution (10^−7^) on LB agar. Addition of SDS or antibiotics reduced the viability of the cells in a strain-dependent manner. In the presence of 1% SDS, the viability of wild type cells was reduced such as growth was only observed up to a dilution of 10^−2^ whereas the ∆*tol*-*pal* mutant did not grow at all. A dramatic decrease in viability of the ∆*tol*-*pal* mutant was also observed in the presence of antibiotics such as 20 µg/ml gentamicin or 100 µg/ml bacitracin. The wild type strain still grew up to a dilution of 10^−7^. In contrast, there were only a few colonies of the ∆*tol*-*pal* mutant at a dilution of 10^−3^ in presence of these antibiotics and even less at higher dilutions. Furthermore, the viability of the ∆*tol*-*pal* mutant was slightly decreased in the presence of colistin in comparison to the wild type. In contrast, there was no difference in viability of the wild type and the ∆*tol*-*pal* mutant in presence of polymyxin B, ampicillin, or streptomycin.

### *A. baumannii* ATCC 19606T ∆*tol-pal* mutant is not impaired in complement resistance

**Fig. 5:**
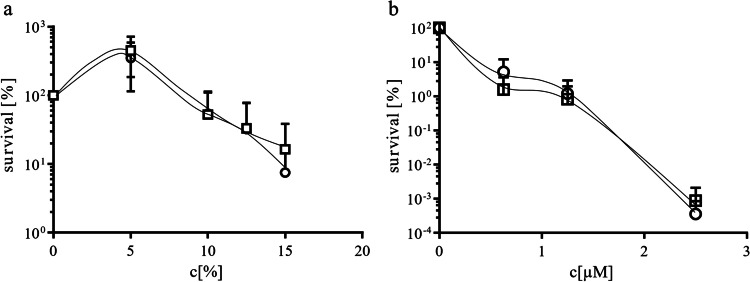
Resistance of the *A. baumannii* wild type strain and the Δ*tol*-*pal* mutant to the human complement system (**a**) and the antimicrobial peptide LL-37 (**b**). The *A. baumannii* wild type strain (□) and the ∆*tol*-*pal* mutant (○) were incubated either with human serum or with the antimicrobial peptide LL-37 at 37 °C, followed by plating onto LB agar to determine the colony forming units. Each value is the mean ± SEM of at least three independent experiments

Next, we analyzed whether the ∆*tol*-*pal* mutant has also a decreased resistance to human antimicrobial compounds. Therefore, we compared the sensitivity of the *A. baumannii* wild type and the ∆*tol*-*pal* mutant to the human complement system (Fig. 5a) and to the antimicrobial human peptide LL-37 (Fig. 5b) which is part of the complement system. Incubation of *A. baumannii* either with human serum or with LL-37 decreased the viability in a concentration-dependent manner. Deletion of the *tol*-*pal* system did not further increase the susceptibility to human serum or LL-37. This suggests that the Tol-Pal core system does not play a role in complement resistance. This is consistent with the results obtained with the *pal* mutant of *E. coli* which also did not show an increased sensitivity to human serum (Diao et al. 2017).

### Tol-Pal system plays an important role in infection of *G. mellonella* larvae

**Fig. 6:**
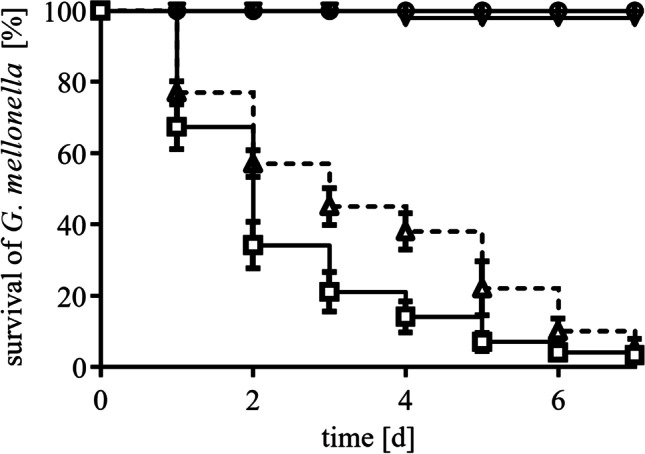
The ∆*tol*-*pal* mutant displays a reduced virulence towards *G. mellonella* larvae. For infection assays, 10 µl of either the *A. baumannii* wild type strain (□) or the Δ*tol*-*pal* (△) mutant (approximately 1*10^6^ CFU) was injected into preselected *G. mellonella* caterpillars followed by incubation at 37 °C for 7 days. A control group was injected with 10 µl sterile saline (▽) and another control group was untreated (○). Each value is the mean ± SEM of at least three independent measurements. The *p* values for the *G. mellonella* survival rates at days 3 and 4 are 0.0138 and 0.0072, respectively

Next, we addressed the role of the Tol-Pal system in virulence of *A. baumannii* by performing *G. mellonella* caterpillar infection studies with the wild type strain and the ∆*tol*-*pal* mutant (Fig. 6). The ∆*tol*-*pal* mutant was reduced in virulence, such as 57% and 45% of the caterpillars infected with the ∆*tol*-*pal* mutant survived after 2 and 3 days, respectively, whereas only 34% and 21% of the larvae survived after 2 and 3 days, respectively, after infection with wild type cells (Fig. 6). This strain dependent difference has been diminished after a prolonged incubation of 5–7 days.

## Discussion

The Tol-Pal system plays an important role in cell division by remodeling septal peptidoglycan at division sites but is also involved in pathogenesis and virulence of pathogenic bacteria (Hirakawa et al. 2020; Webby et al. 2022). Moreover, deletions of Tol-Pal systems induce pleiotropic effects such as release of periplasmic proteins into the extracellular medium and hypersensitivities to detergents and antibiotics (Lazzaroni et al. 1989; Masilamani et al. 2018). The hypersensitivities of the *tol-pal* mutants led to the conclusion that deletion of the Tol-Pal system leads to a disturbed outer membrane cell barrier. This is also consistent with the finding that *tol-pal* deficient bacteria often have an increased sensitivity to surface active compounds, such as SDS or bile salts. In the present study, we identified the Tol-Pal system in *A. baumannii* and report that a ∆*tol*-*pal* mutant exhibits a decreased SDS and antibiotic resistance. This suggests that the Tol-Pal system in *A. baumannii* is also important for membrane integrity and permeability.

The hetero-oligomeric Tol-Pal system of Gram-negative bacteria spans the inner and outer membrane. TolQ, TolR, and TolA are located in the inner membrane and TolB is located in the outer membrane. The lipoprotein Pal is associated with the peptidoglycan and is attached to the outer membrane on the periplasmic side. All Tol-Pal proteins are thought to be involved in energy transduction from the inner to the outer membrane via a cycle of Tol-Pal complex formation and dissociation (Yakhnina and Bernhardt 2020). Thereby the proton motive force at the inner membrane is used. The Tol-Pal dependent energy transduction from the inner to the outer membrane is important for energy driven reactions such as transport through the outer membrane and coordination of peptidoglycan restructuring and septum formation at the division site (Yakhnina and Bernhardt 2020). Our finding that an *A. baumannii ∆tol*-*pal* mutant formed cells with extraordinary length suggests that this mutant is defect in cell division. This is consistent with the findings in *E. coli* where the Tol-Pal system is crucial for efficient cell division, such as deletion of *tol*-*pal* genes resulted in cells with unequal and extended lengths. The same holds true for *Vibrio cholerae* where deletion of *tol* genes led to mutants which formed long filaments with unequal length (Meury and Devilliers 1999; Heilpern and Waldor 2000; Llamas et al. 2000; Tan and Chng 2021).

Our results show that deletion of the Tol-Pal system in *A. baumannii* leads to a decreased killing of *G. mellonella*. This could be due to a decreased secretion of virulence factors or a decreased adherence to host cells. A role of the Tol-Pal system in secretion of virulence factors has already been reported, such as Hirakawa reported that the deletion of *tolB* in enterohemorrhagic *E. coli* (EHEC) decreased the type III secretion system mediated secretion of virulence factors thereby lowering virulence. Moreover, flagella-dependent motility in broth and adhesion to epithelial cells was also impaired (Hirakawa et al. 2020). A role of the Tol-Pal system in virulence has also been reported for *Citrobacter rodentium* which causes lethality in mice, such as a *tolB* mutation of *C. rodentium* abolished lethality in mice (Hirakawa et al. 2020). The authors proposed that the decreased virulence is caused by the dysregulation of the type III secretion system and flagellar activity. The role of the *tol*-*pal* system in virulence of *A. baumannii* has not been elucidated so far. However, our finding that a Δ*tolQ-pal* mutant is impaired in *G. mellonella* killing suggests that the Tol-Pal system is also implicated in virulence of *A. baumannii*. Whether the decreased virulence of the Δ*tolQ-pal* mutant is due to an altered membrane permeability, decreased adhesion to host cells, or even an impaired ability to evade the host defense system will be subject of future studies.

## Conclusions

We have shown here that the Tol-Pal system of *A. baumannii* is required for resistance to antibiotics as well as important for cell morphology and virulence in *G. mellonella* caterpillars. We suggest that the deletion of the Tol-Pal system reduces intrinsic resistance to antibiotics by increasing the membrane permeability, thereby causing an increased influx of antibiotics. Our finding that the ∆*tol*-*pal* mutant was impaired in killing of *G. mellonella* larvae suggests that the Tol-Pal system is also important for virulence of *A. baumannii* and it is tempting to speculate that it plays a role in host cell adhesion and/or evasion of host defense mechanisms.

## Data Availability

Data are available from the authors.
